# Economic Cost of *Campylobacter*, Norovirus and Rotavirus Disease in the United Kingdom

**DOI:** 10.1371/journal.pone.0138526

**Published:** 2016-02-01

**Authors:** Clarence C Tam, Sarah J O’Brien

**Affiliations:** 1 Saw Swee Hock School of Public Health, National University of Singapore, Singapore, Singapore; 2 Faculty of Epidemiology and Population Health, London School of Hygiene & Tropical Medicine, London, United Kingdom; 3 NIHR Health Protection Research Unit in Gastrointestinal Infections, University of Liverpool, Liverpool, United Kingdom; 4 Institute of Infection and Global Health, University of Liverpool, Liverpool, United Kingdom; The Australian National University, AUSTRALIA

## Abstract

**Objectives:**

To estimate the annual cost to patients, the health service and society of infectious intestinal disease (IID) from *Campylobacter*, norovirus and rotavirus.

**Design:**

Secondary data analysis.

**Setting:**

The United Kingdom population, 2008–9.

**Main outcome measures:**

Cases and frequency of health services usage due to these three pathogens; associated healthcare costs; direct, out-of-pocket expenses; indirect costs to patients and caregivers.

**Results:**

The median estimated costs to patients and the health service at 2008–9 prices were: *Campylobacter* £50 million (95% CI: £33m–£75m), norovirus £81 million (95% CI: £63m–£106m), rotavirus £25m (95% CI: £18m–£35m). The costs per case were approximately £30 for norovirus and rotavirus, and £85 for *Campylobacter*. This was mostly borne by patients and caregivers through lost income or out-of-pocket expenditure. The cost of *Campylobacter*-related Guillain-Barré syndrome hospitalisation was £1.26 million (95% CI: £0.4m–£4.2m).

**Conclusions:**

Norovirus causes greater economic burden than *Campylobacter* and rotavirus combined. Efforts to control IID must prioritise norovirus. For *Campylobacter*, estimated costs should be considered in the context of expenditure to control this pathogen in agriculture, food production and retail. Our estimates, prior to routine rotavirus immunisation in the UK, provide a baseline vaccine cost-effectiveness analyses.

## Introduction

Infectious intestinal disease (IID) causes substantial disease burden in both high- and low-income countries. In a population-based study conducted in 2008–9 (IID2), it was estimated that a quarter of the UK population experiences IID annually [1]; norovirus and *Campylobacter*, the most common viral and bacterial pathogens, account for 3 million and 0.5 million annual cases respectively. The sheer volume of IID cases results in considerable, potentially preventable economic burden, both through healthcare use and lost productivity. In a study of the health and economic burden of IID (IID1), conducted in the 1990s, the societal cost, including both costs to the health service and to patients, was estimated to be nearly £750 million annually (1994–5 prices), with the greatest shares coming from *Campylobacter* (£69.6 million), *E*. *coli* (£69.3 million, including non-Shiga toxin-producing strains), *Salmonella* (£46.4 million), norovirus (£24.4 million) and rotavirus (£18.2 million) [2]. These costs are a gross underestimate for norovirus, as current molecular diagnostics are far superior to electron microscopy used in IID1 [3][4]. Overall rates of norovirus disease in IID2, based on polymerase chain reaction (PCR), were 3.7 times higher than those in IID1 [1,5]. Furthermore, changes to the UK health service mean that these earlier estimates may no longer reflect accurately the cost of IID to the health service, or the distribution of cost between the health service and patients; since IID1 was conducted, telephone information and advice lines and out-of-hours services have been introduced, while data from IID2 indicate that IID-related general practice (GP) consultations halved between 1995 and 2009 [1]. Finally, while these costs include only the costs of acute illness, several pathogens also have longer-term sequelae, such as *Campylobacter*-associated Guillain-Barré syndrome [6]. We therefore estimated the societal cost due to three IID pathogens of major public health significance in the UK: *Campylobacter*, norovirus and rotavirus. These pathogens were selected because of their considerable burden, the fact that their incidence has not decreased in the past two decades, and because of their policy relevance. *Campylobacter* and norovirus are both high priority pathogens in the UK Food Standards Agency’s foodborne disease strategy [7], while the recent introduction of routine rotavirus immunisation, and increasing prospects for introduction of norovirus vaccines make these two pathogens very relevant to control policy.

## Materials and Methods

We used data from IID2 to estimate the incidence and health service usage due to these three pathogens in the United Kingdom (comprising England, Wales, Scotland and Northern Ireland) in 2008–9, when IID2 was conducted. The UK population in 2009 was 61.8 million. Health services measured included use of telephone health and advice services; in-person, telephone, and out-of-hours GP consultations; Accident & Emergency department visits; and hospital admissions. Due to uncertainty in estimating hospitalisations, we used three different approaches to estimate the number of hospital admissions for each pathogen. We used NHS reference costs and data from previous studies to estimate costs of illness to patients and the health service. For *Campylobacter*, we additionally estimated the cost of Guillain-Barré syndrome-related hospitalisation, using published GBS incidence data [6].

We expressed costs in monetary terms, because in a universal, provider-pays healthcare system such as the UK National Health Service (NHS), this facilitates comparison of relative costs borne by the health service and patients. A monetary metric also provides baseline data to evaluate the cost-effectiveness of future interventions which, for zoonotic pathogens such as *Campylobacter*, involve costs to other sectors, including agriculture and food production.

### Disease incidence

The methods for IID2 are detailed elsewhere [1]. IID2 measured the rates of (1) sporadic, non-outbreak related IID in a cohort of 6,836 participants (the community cohort), and (2) IID-related consultations in 37 general practice clinics (the GP presentation study). Incidence estimates were standardised to the age and sex distribution of the UK, and calculated for all IID and for 12 individual pathogens. We used these data to derive parameters describing disease incidence and associated uncertainty for each pathogen (see Technical Appendix). We obtained these parameters for the rates of GP consultation and the community incidence, which includes cases who do not consult health services.

### Primary care usage

Primary care services were defined as:

1. In-person GP consultation—a face-to-face consultation with a GP in a clinic
2. Telephone GP consultation—a consultation with a GP over the telephone
3. Out-of-hours consultation—a consultation with a dedicated service providing health services outside GP operating hours
4. Accident & Emergency visit—a consultation at a hospital Accident & Emergency department
5. Telephone information and advice line—a telephone call to a dedicated service providing syndrome-based medical triage and advice (at the time IID2, these services were NHS Direct in England and Wales, and NHS24 in Scotland)

We used data from GP presentation study cases to estimate pathogen-specific usage of in-person, telephone and out-of-hours consultations. We employed data from cases in the community cohort to estimate usage of telephone information and advice lines, and visits to Accident & Emergency departments. As the number of cases for each pathogen in the community cohort was modest and usage of these services was low, we estimated usage among all IID cases in the cohort rather than by individual pathogen. We modelled the proportion of patients using each service using Beta distributions. The derivation of these Beta distributions and a full description of model parameters, parameter distributions and data sources is provided in the S1 File.

### Hospital admissions

A hospital admission was defined as an overnight stay in a hospital due to illness. Possible sources of hospitalisation data include Hospital Episodes Statistics (HES)—which capture all public hospital admissions by International Classification of Diseases (ICD) code [8]—data on outbreak-related hospitalisations from outbreak surveillance (Gsurv) [9], and cohort study data. HES data have national coverage, but pathogen aetiology is often missing. We used three different scenarios to model pathogen-specific hospitalisations. In scenario 1, we used data on *Campylobacter*, norovirus and rotavirus outbreaks reported to Public Health England between 2000 and 2008 to estimate the proportion of outbreak cases that are hospitalised [9]. Hospitalisation parameters were derived by fitting Beta distributions to these data.

In scenario 2, we pooled data from the GP presentation study components of IID1 and IID2 and derived Beta distributions for the proportion of cases due to each pathogen that were hospitalised.

Scenario 3 differed for each pathogen. For *Campylobacter*, we took simply the number of hospital admissions recorded in Hospital Episode Statistics [8], which compiles data on all in-patient admissions to NHS hospitals. For norovirus, we used estimates of hospitalisations among adults and the elderly, based on data from Haustein et al. [10]. For rotavirus, we used estimates of hospitalisations among children <5 years, based on data from Harris et al. [11].

### Cost data

We obtained costs of GP face-to-face and telephone consultations and visits to Accident & Emergency departments from the Unit Costs of Health and Social Care 2009 [12]. An out-of-hours consultation was priced according to a published study of salmonellosis economic burden [13], adjusted to 2008–9 prices using the Hospital and Community Health Services Pay and Prices Index (HCHS PPI) [12]. The average cost of a call to a telephone information and advice line was based on that reported by Munro [14], as previously described by Harris et al. [11] and updated to 2008–9 prices using the HCHS PPI. For in-patient admissions, the unit cost was based on the average cost of treating a patient with a code of “Infectious or non-infectious gastroenteritis” (currency codes PA21A and PA21B) as reported in the NHS Reference Costs 2008–9 [15].

We used detailed economic data collected from cases in IID1 to estimate costs to patients. These included direct, out-of-pocket expenses resulting from illness, such as medications, transport to health clinics, childcare, cleaning products, special foods etc., and indirect costs of lost income by patients themselves or by caregivers [2,16]. We obtained these costs separately for patients who used health services and those who did not, and updated them to 2008–9 prices using the Retail Price Index (RPI).

### *Campylobacter*-associated GBS admissions

We previously estimated that 1 in 5,000 campylobacteriosis cases develop GBS [6]. We applied this fraction to the annual expected cases of campylobacteriosis to estimate the number of hospitalisations for *Campylobacter*-related GBS. We used data from HES for ICD 10^th^ Revision (ICD10) code G61.0 (Guillain-Barré syndrome) to determine the proportion of GBS hospitalisations that arise from emergency, elective and day case admissions. We estimated costs for different types of admissions using NHS reference costs based on Healthcare Resource Group (HRG) codes for Neurological System Disorders with and without complications (PA01A and PA01B respectively). Because GBS patients are admitted for an average of 30 days, we estimated the average cost of one hospital-day for emergency and elective admissions and multiplied this by the average duration of a GBS hospitalisation to obtain the relevant unit costs. We multiplied each unit cost by the number of emergency, elective and day case admissions respectively, and summed these products to obtain the total cost of *Campylobacter*-related GBS hospitalisation.

### Cost model

For each pathogen, we estimated the number of cases using each type of health service by multiplying the expected number of cases by the estimated proportion using each service. We accounted for uncertainty by drawing at random from the distribution of each estimated parameter described above. We used the median of 9,999 simulations as the point estimate and the 2.5^th^ and 97.5^th^ percentiles as the lower and upper 95% confidence bounds. We then multiplied the number of cases using each service by its corresponding unit cost to estimate the total cost of each service and summed these totals to obtain the overall cost of healthcare due to each pathogen.

To estimate costs to patients, we multiplied the average cost per case (including both direct and indirect costs) by the number of cases, separately for those who used and did not use health services. The sum of these products was the overall cost to patients.

We estimated the societal cost by summing healthcare and patient costs. For each pathogen, we obtained the average cost per case by dividing the societal cost by the number of cases.

Analysis was conducted using Stata 13.0 (Stata Corporation) and Microsoft Excel 2010. A detailed description of the model parameters for each pathogen is given in Tables A–C of S1 File. Plots of fitted parameter distributions for each pathogen are given in Figs A–C of S1 File. The unit cost of each health service is given in Table D of S1 File.

### Ethics statement

No institutional review was required for this secondary data analysis.

## Results

The total costs to patients were £45.3 million for *Campylobacter*, £69.3 million for norovirus and £14.1 million for rotavirus. Point estimates of healthcare costs, including primary care and hospitalisations, ranged between £4.2–£5.1 million for *Campylobacter*, £11.9 –£17.7 million for norovirus and £5.1–£10.9 million for rotavirus, depending on the method for estimating hospitalisation costs.

### Cases, primary care consultations and hospitalisations

**Fig 1:**
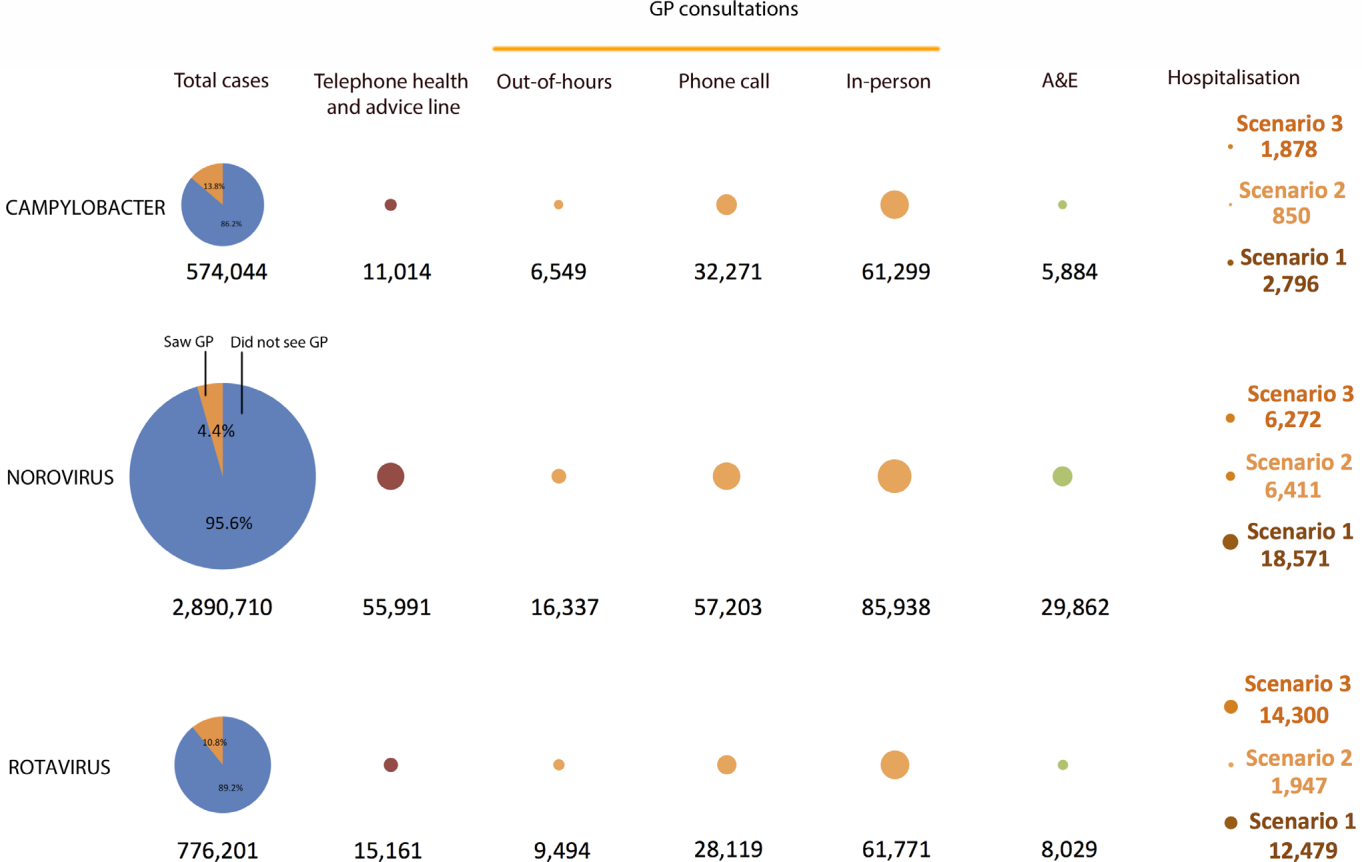
Estimated cases, healthcare consultations and hospitalisations due to *Campylobacter*, norovirus and rotavirus disease in the United Kingdom, 2008–9. Area of circles is proportional to the estimated number of cases.

Estimated cases, primary care consultations and hospitalisations for each pathogen are shown in Table 1, together with associated costs. Hospitalisation estimates differed considerably between scenarios; estimates from outbreak data (scenario 1) were higher than cohort estimates for all pathogens (scenario 2). For norovirus, the latter were similar to estimates from Haustein for adults and the elderly, while for rotavirus, estimates from outbreak data were similar to those from Harris for children <5 years. Primary care usage was similar for *Campylobacter* and rotavirus IID cases, with the latter showing somewhat higher use of telephone information and advice lines, out-of-hours clinics, and Accident & Emergency services (Fig 1). Among norovirus cases, only 4.4% consulted a GP, but the large number of cases means that norovirus accounted for more primary care consultations than either *Campylobacter* or rotavirus.

**Table 1:** Estimated cases, healthcare usage and societal costs of IID associated with *Campylobacter*, norovirus and rotavirus in the UK, 2008–9.

*Health service costs*	Cases	(95% CI)	Unit cost	Estimated cost	(95% CI)
*CAMPYLOBACTER*					
Telephone information and advice line	11,014	(6,722–17,960)	£20.53	£226,124	(£138,010–£368,739)
Out-of-hours consultation	6,549	(3,633–10,947)	£77.92	£510,248	(£283,072–£852,977)
Phone call to GP	32,271	(22,791–45,430)	£18.00	£580,886	(£410,238–£817,746)
In-person GP consultation	61,299	(45,106–83,079)	£31.00	£1,900,277	(£1,398,294–£2,575,451)
Accident & Emergency	5,884	(3,237–10,271)	£93.00	£547,226	(£301,020–£955,192)
Hospitalisation					
*Scenario 1*	2,796	(842–7,111)	£467.09	£1,305,907	(£393,213–£3,321,326)
*Scenario 2*	850	(271–2,036)	£467.09	£396,909	(£126,496–£951,215)
*Scenario 3*	1,878		£467.09	£877,188	(£877,188–£877,188)
		*Total health service cost*	*Scenario 1*	*£5,070,668*	(*£2,923,848–£8,891,430*)
			*Scenario 2*	*£4,161,670*	(*£2,657,130–£6,521,320*)
			*Scenario 3*	*£4,641,949*	(*£3,407,822–£6,447,292*)
Cost to patients					
*Not seeking medical care*	495,071	(288,945–803,770)	£30.99	£15,340,756	(£8,953,529–£24,906,430)
*Seeking medical care*	78,973	(55,486–112,401)	£379.61	£29,979,318	(£21,063,306–£42,669,081)
		*Total cost to patients*		*£45,320,074*	(*£30,016,835–£67,575,511*)
		**Total societal cost**	***Scenario 1***	**£50,390,742**	(**£32,940,683–£76,466,942**)
			***Scenario 2***	**£49,481,744**	(**£32,673,965–£74,096,831**)
			***Scenario 3***	**£49,962,023**	(**£33,424,657–£74,022,803**)
*NOROVIRUS*					
Telephone information and advice line	55,991	(38,860–79,174)	£22.50	£1,259,804	(£874,358–£1,781,406)
Out-of-hours consultation	16,337	(9,673–26,412)	£77.92	£1,272,934	(£753,676–£2,058,006)
Phone call to GP	57,203	(39,866–81,133)	£18.00	£1,029,658	(£717,587–£1,460,402)
In-person GP consultation	85,938	(61,640–118,510)	£31.00	£2,664,085	(£1,910,834–£3,673,801)
Accident & Emergency	29,862	(18,027–47,013)	£93.00	£2,777,150	(£1,676,511–£4,372,245)
Hospitalisation					
*Scenario 1*	18,571	(12,823–26,152)	£467.09	£8,674,167	(£5,989,310–£12,215,441)
*Scenario 2*	6,411	(1,993–14,939)	£467.09	£2,994,607	(£930,861–£6,978,019)
*Scenario 3*	6,272	(5,165–7,379)	£467.09	£2,929,601	(£2,412,374–£3,446,828)
		*Total health service cost*	*Scenario 1*	*£17,677,798*	(*£11,922,276–£25,561,301*)
			*Scenario 2*	*£11,998,238*	(*£6,863,827–£20,323,879*)
			*Scenario 3*	*£11,933,232*	(*£8,345,340–£16,792,688*)
Cost to patients					
*Not seeking medical care*	2,762,688	(2,290,887–3,314,740)	£15.82	£43,700,199	(£36,237,251–£52,432,557)
*Seeking medical care*	128,022	(88,784–184,600)	£199.68	£25,563,266	(£17,728,273–£36,860,687)
		*Total cost to patients*		*£69,263,465*	(*£53,965,524–£89,293,245*)
		**Total societal cost**	***Scenario 1***	**£86,941,263**	(**£65,887,800–£114,854,545**)
			***Scenario 2***	**£81,261,703**	(**£60,829,350–£109,617,123**)
			***Scenario 3***	**£81,196,697**	(**£62,310,864–£106,085,932**)
*ROTAVIRUS*					
Telephone information and advice line	15,161	(9,568–23,563)	£20.53	£311,279	(£196,450–£483,783)
Out-of-hours consultation	9,494	(4,482–17,850)	£77.92	£739,724	(£349,230–£1,390,855)
Phone call to GP	28,119	(17,885–43,584)	£18.00	£506,143	(£321,938–£784,518)
In-person GP consultation	61,771	(42,897–89,415)	£31.00	£1,914,900	(£1,329,812–£2,771,858)
Accident & Emergency	8,029	(4,495–13,625)	£93.00	£746,668	(£418,024–£1,267,100)
Hospitalisation					
*Scenario 1*	12,479	(6,553–21,953)	£467.09	£5,828,683	(£3,060,901–£10,254,077)
*Scenario 2*	1,947	(642–4,542)	£467.09	£909,220	(£299,861–£2,121,689)
*Scenario 3*	14,300	(13,472–15,160)	£467.09	£6,679,331	(£6,292,554–£7,080,983)
		*Total health service cost*	*Scenario 1*	*£10,047,399*	(*£5,676,355–£16,952,191*)
			*Scenario 2*	*£5,127,936*	(*£2,915,315–£8,819,803*)
			*Scenario 3*	*£10,898,047*	(*£8,908,009–£13,779,097*)
Cost to patients					
*Not seeking medical care*	692,351	(447,160–1,046,618)	£0.00	£0.00	—
*Seeking medical care*	83,850	(54,868–128,141)	£168.27	£14,109,549	(£9,232,710–£21,562,453)
		*Total cost to patients*		*£14,109,549*	(*£9,232,710–£21,562,453*)
		**Total societal cost**	***Scenario 1***	**£24,156,948**	(**£14,909,065–£38,514,644**)
			***Scenario 2***	**£19,237,485**	(**£12,148,025–£30,382,256**)
			***Scenario 3***	**£25,007,596**	(**£18,140,719–£35,341,550**)

Footnote for Table 1: Seeking medical care is defined as using a health service as a result of illness. Health services are defined as follows: In-person GP consultation—a face-to-face consultation with a GP that takes place at the clinic; Telephone GP consultation—a consultation with a GP that takes place over the telephone; Out-of-hours consultation—a consultation with a dedicated service that provides health services outside GP operating hours (evenings and weekends); Accident & Emergency visit—a consultation at a hospital Accident & Emergency department; Telephone information and advice line—a telephone call to a dedicated service that provides syndrome-based medical triage and advice (at the time of this study, these services were NHS Direct in England and Wales, and NHS24 in Scotland); Hospital admission—an overnight stay in a hospital as a result of illness.

The number of hospitalisations due to rotavirus was about double that for norovirus (based on scenarios 2 and 3) and 5–6 times higher than for *Campylobacter* (based on scenarios 1 and 3).

### Cost to patients and the health service

Overall costs of acute illness due to *Campylobacter* were approximately £50 million. For norovirus, total costs exceeded £80 million. The choice of hospitalisation scenario had little impact on overall costs for these two pathogens. Rotavirus costs varied between £20-£25 million and were more sensitive to the choice of hospitalisation scenario, because hospitalisation rates for this pathogen were higher.

**Fig 2:**
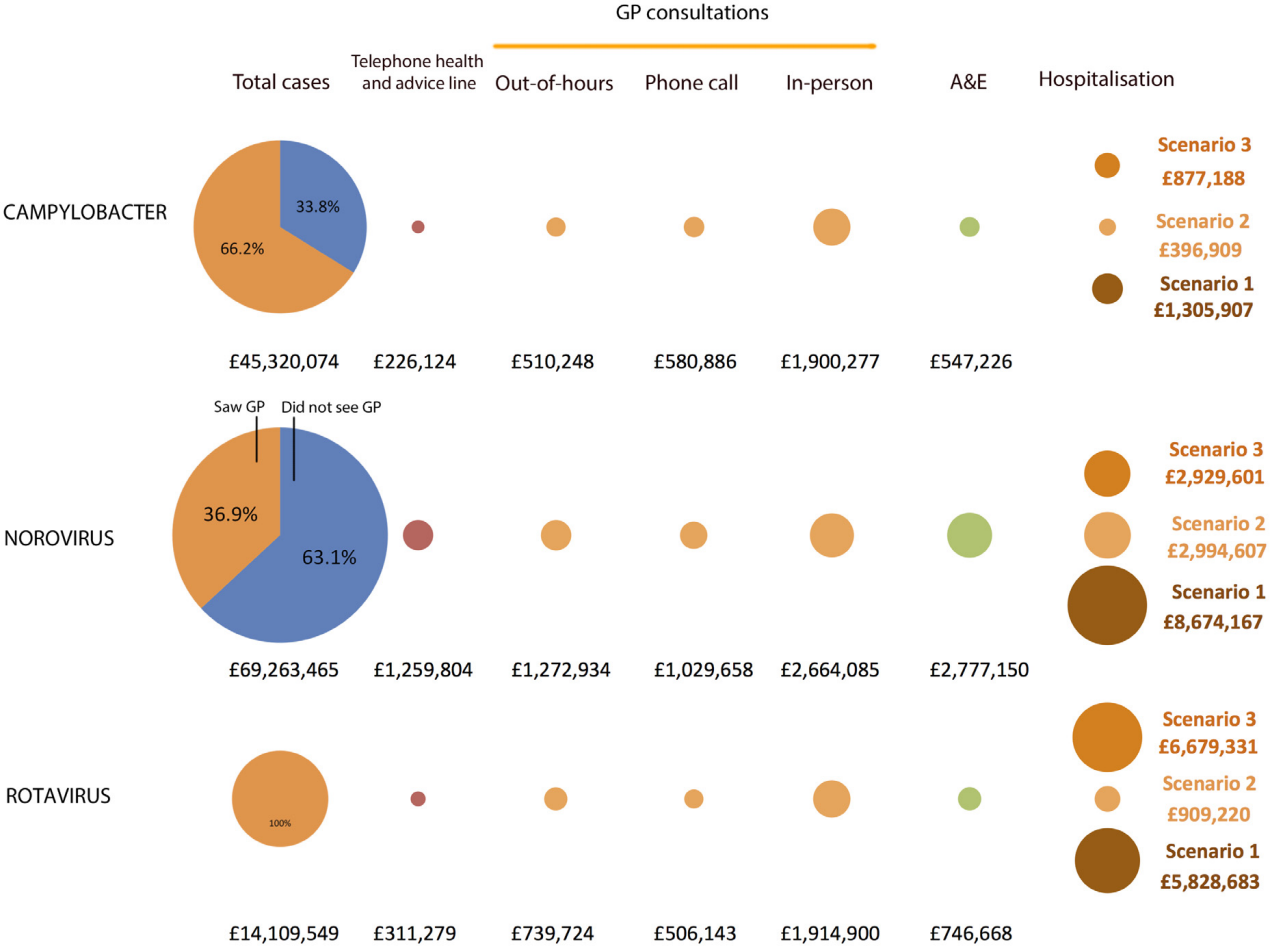
Estimated costs to patients and the health service due to *Campylobacter*, norovirus and rotavirus disease in the United Kingdom, 2008–9. Area of circles is proportional to the estimated cost

Costs to patients and different parts of the health sector are shown in Fig 2. *Campylobacter* and norovirus accounted for £45 million and £69 million in patient costs respectively. For *Campylobacter*, 66% of this cost was borne by patients seeking healthcare, likely reflecting greater illness severity relative to norovirus, for which 37% of patient costs were associated with patients who consulted their GP. For all three pathogens, costs to patients greatly exceeded costs to the health sector; for *Campylobacter*, patient costs exceeded health service costs by 10-fold. Norovirus accounted for much higher healthcare costs than the other two pathogens, particularly in terms of consultations to telephone information and advice lines, A&E visits and hospitalisations.

The average cost per case of acute illness was approximately £30 for both norovirus and rotavirus. For *Campylobacter* this rose to more than £85. Table E in S1 File shows the cost per case for each pathogen, broken down into direct costs to patients and loss of employment in patients and carers, both for cases that resulted in medical consultations and cases that did not involve healthcare usage.

### Costs of *Campylobacter*-associated GBS

There were an estimated 112 annual GBS cases associated with *Campylobacter* IID. The average cost per GBS case was £11,296.50. GBS added a further £1.27 million to *Campylobacter*-related hospitalisation costs (95% CI: £0.4m–£4.2m), though uncertainty around this estimate was high due to the rarity of this condition.

## Discussion

We estimate that the economic cost of *Campylobacter*, norovirus and rotavirus disease in the UK exceeds £150 million annually; 80% of this cost is borne by patients, through lost employment and out-of-pocket expenses resulting from illness. The annual cost of norovirus disease exceeds £80 million. Sporadic norovirus disease accounts for more healthcare consultations than either *Campylobacter* or rotavirus, while previous work indicates that norovirus outbreaks in healthcare settings cost the NHS over £100 million annually (2002–3 prices) [17].

Our work makes several improvements on previous estimates. Previous UK rotavirus estimates included only hospitalisation costs [18,19], total healthcare costs [11], or societal costs from patients <5 years seeking healthcare [20]. Only IID1 included costs to patients not seeking healthcare, but that study lacked data to estimate hospitalisation costs [2]. Our estimates comprise costs to the NHS and to patients, including those not seeking healthcare. Further, our study provides the first reliable estimates of the societal cost of non-outbreak related norovirus, using empirical incidence data and modern diagnostics. Our estimates are three-fold higher higher than previously reported [2].

Wherever possible, we used pathogen-specific incidence and healthcare usage data, except for telephone information and advice consultations, for which data were sparse. We also accounted for uncertainty in incidence estimates. For hospitalisations, we used three different estimation methods for each pathogen. Hospitalisation estimates from outbreak data were higher than cohort-based estimates from IID1 and IID2; it is likely that there were insufficient cases in IID1 and IID2 to reliably estimate hospitalisation. The pathogen-specific epidemiologies should also be considered. For rotavirus, hospitalisation estimates from outbreak data were comparable with those from Harris et al. among children <5 years [11], in whom most hospitalisations occur. For norovirus, however, estimates from outbreak data were considerably higher than other data sources. Most reported norovirus outbreaks occur in institutional settings and involve vulnerable patients with higher hospitalisation risk. Estimates from longitudinal studies (scenario 2) are likely to be more reliable, and these were similar to those of Haustein among adults and the elderly [10]. This also implies that the hospitalisation burden from norovirus falls primarily on adults and the elderly rather than children. For rotavirus, the converse is true, and the total cost was more sensitive to the choice of hospitalisation scenario, because healthcare-associated costs comprise a greater fraction of total costs compared with *Campylobacter* and norovirus.

We estimated the cost of *Campylobacter*-associated GBS hospitalisation at £11,000 per case. This is an underestimate, as it excludes long-term disability costs. Around 15% of GBS patients experience persistent disability at one year [21], around a third have restricted mobility three to six years after onset, and around 40% have to change jobs and alter leisure activities [22]. In addition, NHS reference costs capture average costs for patients undergoing similar procedures, but GBS patients may require more intensive treatment. We addressed this by accounting for longer average duration of GBS hospitalisation and case mix (emergency, elective and day case); previous work indicates that GBS hospitalisation costs correlate strongly with hospitalisation duration [23].

Our study has several limitations. First, we considered costs borne by the NHS and by patients, but not wider societal costs from mortality, illness in caregivers, and costs of lost productivity to employers and the wider economy. Data on these additional costs are currently lacking. Rotavirus mortality is believed to be rare, and has not been considered in previous UK analyses [11]. Harris et al. estimated 85 annual norovirus-associated deaths in UK elderly. We are not aware of similar estimates for *Campylobacter*. Scandinavian studies have described excess mortality among *Campylobacter* patients up to one year post-infection, but these estimates are difficult to generalise to the UK, because of differences in reporting systems and distribution of population risk factors. Further, attributing deaths to gastrointestinal pathogens is problematic, because deaths occur primarily in vulnerable patients with pre-existing conditions. Thus, in some cases an IID pathogen may be causally related to mortality, in others infection will be coincidental, while in others it may precipitate deaths that would eventually have occurred even in the absence of infection.

We did not explicitly take account of hospitalisation duration in our cost estimates. The NHS reference cost for a hospitalisation due to “infectious or non-infectious gastroenteritis” is based on the average duration of hospital stay for a patient with this code, which is 1 day. Hospitalisation data suggest that average length of hospitalisation is longer for *Campylobacter* compared with rotavirus (6 days vs 2 days) [8]. However, inferring differences in hospitalisation duration from these data is problematic. Average hospitalisation duration for *Campylobacter* might also be influenced by sequelae such as Guillain-Barré syndrome. Secondly, average hospitalisation duration among recorded admissions for norovirus is 14 days [8]. However, hospitalised patients with diagnosed rotavirus and particularly norovirus infections represent only a fraction of all hospitalisations from these pathogens—previous work indicates that a large fraction of these are recorded as unspecified viral infections (see our references 10 and 11), which have an average hospitalisation of 1 day. This suggests that viral infections are more likely to be diagnosed in patients with more severe illness or with underlying conditions, and adjusting for length of stay using these data risks grossly overestimating hospitalisation costs. For this reason, we have decided to follow the methodology used in previous economic costing studies for rotavirus in the UK, which have not adjusted further for length of hospitalisation [11]. For *Campylobacter*, it is possible that our conservative approach underestimates hospitalisation costs. However, hospitalisation due to *Campylobacter* is uncommon; even assuming that hospitalisation costs increased by 5-fold, the result would be to double the health service costs. Qualitatively, our results would not change dramatically; the cost to patients would still dominate the overall economic burden.

Our analysis excludes costs from long-term disability. While we included costs of *Campylobacter*-related GBS hospitalisation, data on GBS rehabilitation costs were unavailable. *Campylobacter* can cause other sequelae, including reactive arthritis in approximately 1% cases [24], and irritable bowel syndrome in approximately 6% of patients [25]. Again, data on costs associated with these conditions were unavailable.

We estimated costs across all age groups, although health service costs could vary by age group. However, NHS reference costs are not stratified by age group. Further, they do not include information on variability in healthcare costs. As with other previous studies [11], we could not account for uncertainty in treatment costs.

We used the best available data sources, but some of our estimates are subject to considerable uncertainty due to sparse data, particularly for both hospitalisation costs and costs to patients. For rotavirus, we assumed that the cost to patients not seeking healthcare is zero; IID1 included economic data from a small number of rotavirus patients, all of whom sought healthcare. Patient costs due to rotavirus are therefore underestimated.

Overall societal costs of *Campylobacter* disease in our study (£50 million) were lower than in IID1 (£70 million) [2], although campylobacteriosis incidence has remained stable [1,5]. This decrease in cost results from reductions in GP usage; IID-related GP consultation rates halved between 1995 and 2009 [1]. This has also reduced the cost per case (from £164 in IID1 to £85 here). Our estimates, however, should be considered in the context of expenditure to control *Campylobacter*. These costs fall under other economic sectors, including agriculture, food production and retail, and are poorly characterised. Studies considering the cost of such expenditures relative to the cost of illness averted are important to evaluate the cost-effectiveness of *Campylobacter* interventions, which will be useful for guiding control policies in addition to the wider socio-political and public health considerations. For norovirus, the improved ascertainment of cases in IID2, most of whom do not seek healthcare, has resulted in much higher estimates of overall cost, while the cost per case is 30% lower than previously reported in IID1. For rotavirus, overall costs are somewhat higher (£25 million vs £18 million) due to increases in incidence since IID1, although the cost per case is about 45% lower. These costs will continue to change over time, following the recent introduction of rotavirus vaccination into the UK immunisation programme. Our results therefore provide a baseline with which to evaluate the cost effectiveness of rotavirus immunisation in future.

## Supporting Information

S1 File Economic cost of *Campylobacter*, norovirus and rotavirus disease in the United Kingdom: Technical appendix. (DOCX)

S2 File EVEREST checklist. (DOC)
