# Supplementary material for: Economic Cost of Campylobacter, Norovirus and Rotavirus Disease in the United Kingdom
Source: PLoS One. 2016 Feb 1;11(2):e0138526. doi: 10.1371/journal.pone.0138526 (PMC4735491; doi:10.1371/journal.pone.0138526)
Supplement: S1 File — (DOCX) [file pone.0138526.s001.docx]

# Economic cost of *Campylobacter*, norovirus and rotavirus disease in the United Kingdom: Technical appendix

## Description of data sources

### IID2 Cohort Study (2008-9)

6836 individuals of all ages were recruited from the population registered with 88 general practices in the UK. Participants reported on a weekly basis whether or not they had symptoms of IID for a period of up to 52 weeks. IID cases completed a questionnaire enquiring about symptoms and usage of health services, including telephone health and advice services; general practice consultations in person, by telephone, and out of hours; visits to Accident & Emergency departments; and admissions to hospital. IID cases also submitted a stool specimen for microbiological confirmation. The study comprised 4658 person-years of follow-up and measured the population incidence of IID overall and for 12 individual pathogens [1].

### IID2 GP Presentation Study (2008-9)

Patients presenting with IID were recruited from 37 general practices in the UK. IID cases completed a questionnaire enquiring about symptoms and usage of health services, including telephone health and advice services; general practice consultations in person, by telephone, and out of hours; visits to Accident & Emergency departments; and admissions to hospital. IID cases also submitted a stool specimen for microbiological confirmation. The study comprised 312,232 person-years of follow-up and measured IID primary care consultation rates overall and for 12 individual pathogens [1].

### IID Study (1993-6)

This study comprised Cohort and GP Presentation studies using similar methodologies as described above for the IID2 Study. In addition, the IID Study included an economic component: IID cases were interviewed using an economic questionnaire to obtain information about health services used and costs incurred as a result of illness. Costs to patients and their carers included lost wages due to absence from work, costs related to healthcare consulation (transportation, prescription medication, telephone charges) and other costs incurred as a direct result of illness (special foods, purchase of additional hygiene or cleaning products, cancellation of pre-arranged activities, childcare arrangements). Costs were estimated for all IID and for specific pathogens [2].

### Outbreak surveillance (GSurv)

Surveillance of IID-related outbreaks has been conducted by Public Health England (PHE) since 1992. Outbreaks are reported and investigated by local or regional Medical Officers of Health, and a minimum dataset for each outbreak is returned to PHE at the end of the investigation. This minimum dataset includes information on the outbreak setting, the mode(s) of transmission, the number of cases affected, the number of cases hospitalised, the number of deaths, details of investigations conducted, the suspected vehicle of infection, the suspected pathogen, and the type of evidence to support the pathogen and vehicle suspected [3].

## Incidence model

We used the model below to estimate the number of cases, general practice consultations and health service usage for the three pathogens *Campylobacter*, norovirus and rotavirus.

*C_p_ = N * c_p_*

*G_p_ = N * g_p_*

*A_p_ = C_p_ * a*

*E_p_ = C_p_ * e*

*O_p_ = G_p_ * o_p_*

*T_p_ = G_p_ * t_p_*

*I_p_ = G_p_ * i_p_*

*H_p_ = C_p_ * h_p_*

*S_campy_ = C_p_ * s_campy_*

| Parameter | Description |
| --- | --- |
| *N* | UK population in 2009 = 61,792,000 |
| *C_p_* | Total IID cases due to pathogen *p* |
| *c_p_* | Population rate of IID due to pathogen *p* |
| *G_p_* | Total general practice consultations due to pathogen *p* |
| *g_p_* | Rate of consultation to general practice for IID due to pathogen *p* |
| *A_p_* | Calls to telephone health and advice services due to pathogen *p* |
| *a* | Proportion of IID cases in the IID2 Cohort Study using telephone health and advice services (all pathogens) |
| *E_p_* | Visits to Accident & Emergency departments for IID due to pathogen *p* |
| *e* | Proportion of IID cases in the IID2 Cohort Study visiting Accident & Emergency departments (all pathogens) |
| *O_p_* | Out-of-hours consultations to general practice due to pathogen *p* |
| *o_p_* | Proportion of IID cases in the IID2 GP Presentation Study due to pathogen *p* that consulted out-of-hours services |
| *T_p_* | Telephone consultations to general practice due to pathogen *p* |
| *t_p_* | Proportion of IID cases in the IID2 GP Presentation Study due to pathogen p that consulted their general practice by telephone |
| *I_p_* | In-person general practice consultations due to pathogen *p* |
| *i_p_* | Proportion of IID cases in the IID2 GP Presentation Study due to pathogen *p* that consulted their general practice in person |
| *H_p_* | Hospital admissions due to pathogen *p* |
| *h_p_* | Proportion of IID cases due to pathogen *p* that were hospitalised |
| *S_campy_* | Hospitalisations for Guillain-Barré syndrome due to *Campylobacter* |
| *s_campy_* | Rate of Guillain-Barré syndrome among *Campylobacter* IID cases |

The parameter distributions for each pathogen are described in Annex Tables 1-3. Rate parameters were modelled using Normal distributions defined by the log rate and the corresponding standard deivation. Proportions were modelled using Beta distributions. Parameters for the Beta distributions were obtained by fitting a Beta function to the observed data using maximum likelihood methods. The resulting distributions were checked visually as shown in Appendix Figures 1-3. No acceptance criteria were used.

For each outcome, estimated frequencies were obtained by drawing parameter values at random from the relevant distributions. The median from 9999 simulations was taken as the point estimate, and the 2.5^th^ and 97.5^th^ centiles as the lower and upper 95% confidence bounds.

Analysis was conducted in Stata 13.0 (Stata Corporation).

## Table A: Model parameters for *Campylobacter*

|  | Data | | | Model |  |  |
| --- | --- | --- | --- | --- | --- | --- |
| Parameter | Mean | Denominator | Denominator units | Distribution | Variable | Data source |
| *c_p_* | 0.00951 | 4658.6 | Person-years | N(-4.68,0.22) | Population rate | IID2 Cohort Study [1] |
| *g_p_* | 0.00130 | 312,232 | Person-years | N(-6.66,0.18) | GP consultation rate | IID2 GP Presentation Study [1] |
| *a* | 0.0197 | 1323 | Cases | Beta(25.8,1289.0) | Telephone health and advice line | IID2 Cohort Study |
| *o_p_* | 0.0850 | 142 | Cases | Beta(11.6,124.1) | Out-of-hours consultation | IID2 GP Presentation Study |
| *t_p_* | 0.409 | 142 | Cases | Beta(57.4,82.9) | GP telephone consultation | IID2 GP Presentation Study |
| *i_p_* | 0.775 | 142 | Cases | Beta(107.9,31.4) | GP in-person consultation | IID2 GP Presentation Study |
| *e* | 0.0106 | 1318 | Cases | Beta(13.1,1228.9) | Accident & Emergency visits | IID2 Cohort Study |
| *h_p1_* | 0.00549 | 424 | Cases | Beta(3.1,558.7) | Hospitalisation (scenario 1) | Outbreak data (Gsurv) |
| *h_p2_* | 0.00164 | 441 | Cases | Beta(3.5,2119.3) | Hospitalisation (scenario 2) | Combined IID1 & IID2 GP Presentation Study data |
| *s_campy_* | 0.00117 | 2560 | Person-years | N(-6.75,0.574) | Rate of Guillain-Barré syndrome among *Campylobacter* IID cases | General Practice Research Database [4] |

Health services are defined as follows: In-person GP consultation – a face-to-face consultation with a GP that takes place at the clinic; Telephone GP consultation – a consultation with a GP that takes place over the telephone; Out-of-hours consultation – a consultation with a dedicated service that provides health services outside GP operating hours (evenings and weekends); Accident & Emergency visit – a consultation at a hospital Accident & Emergency department; Telephone information and advice line – a telephone call to a dedicated service that provides syndrome-based medical triage and advice (at the time of this study, these services were NHS Direct in England and Wales, and NHS24 in Scotland); Hospital admission – an overnight stay in a hospital as a result of illness.

## Table B: Model parameters for norovirus

|  | Data | | | Model |  |  |
| --- | --- | --- | --- | --- | --- | --- |
| Parameter | Mean | Denominator | Denominator units | Distribution | Variable | Data source |
| *c_p_* | 0.0471 | 4658.6 | Person-years | N(-3.06,0.09) | Population rate | IID2 Cohort Study [1] |
| *g_p_* | 0.00210 | 312,232 | Person-years | N(-6.18,0.19) | GP consultation rate | IID2 GP Presentation Study [1] |
| *a* | 0.0197 | 1323 | Cases | Beta(25.3,1262.4) | Telephone health and advice line | IID2 Cohort Study |
| *o_p_* | 0.131 | 122 | Cases | Beta(15.5,102.8) | Out-of-hours consultation | IID2 GP Presentation Study |
| *t_p_* | 0.451 | 122 | Cases | Beta(53.4,65.1) | GP telephone consultation | IID2 GP Presentation Study |
| *i_p_* | 0.672 | 122 | Cases | Beta(82.9,40.6) | GP in-person consultation | IID2 GP Presentation Study |
| *e* | 0.0106 | 1318 | Cases | Beta(13.6,1265.1) | Accident & Emergency visits | IID2 Cohort Study |
| *h_p1_* | 0.00649 | 12333 | Cases | Beta(26.4,4037.4) | Hospitalisation (scenario 1) | Outbreak data (Gsurv) |
| *h_p2_* | 0.00246 | 201 | Cases | Beta(3.2,1295.6) | Hospitalisation (scenario 2) | Combined IID1 & IID2 GP Presentation Study data |

Health services are defined as follows: In-person GP consultation – a face-to-face consultation with a GP that takes place at the clinic; Telephone GP consultation – a consultation with a GP that takes place over the telephone; Out-of-hours consultation – a consultation with a dedicated service that provides health services outside GP operating hours (evenings and weekends); Accident & Emergency visit – a consultation at a hospital Accident & Emergency department; Telephone information and advice line – a telephone call to a dedicated service that provides syndrome-based medical triage and advice (at the time of this study, these services were NHS Direct in England and Wales, and NHS24 in Scotland); Hospital admission – an overnight stay in a hospital as a result of illness.

## Table C: Model parameters for rotavirus

|  | Data | | | Model |  |  |
| --- | --- | --- | --- | --- | --- | --- |
| Parameter | Mean | Denominator | Denominator units | Distribution | Variable | Data source |
| *c_p_* | 0.0129 | 4658.6 | Person-years | N(-4.37,0.19) | Population rate | IID2 Cohort Study [1] |
| *g_p_* | 0.00139 | 312,232 | Person-years | N(-6.6,0.21) | GP consultation rate | IID2 GP Presentation Study [1] |
| *a* | 0.0197 | 1323 | Cases | Beta(25.7,1274.3) | Telephone health and advice line | IID2 Cohort Study |
| *o_p_* | 0.118 | 68 | Cases | Beta(7.1,53.1) | Out-of-hours consultation | IID2 GP Presentation Study |
| *t_p_* | 0.338 | 68 | Cases | Beta(22.5,43.9) | GP telephone consultation | IID2 GP Presentation Study |
| *i_p_* | 0.735 | 68 | Cases | Beta(47.8,17.2) | GP in-person consultation | IID2 GP Presentation Study |
| *e* | 0.0106 | 1318 | Cases | Beta(12.9,1207.0) | Accident & Emergency visits | IID2 Cohort Study |
| *h_p1_* | 0.0166 | 1211 | Cases | Beta(10.6,624.3) | Hospitalisation (scenario 1) | Outbreak data (Gsurv) |
| *h_p2_* | 0.00276 | 64 | Cases | Beta(3.6,1295.6) | Hospitalisation (scenario 2) | Combined IID1 & IID2 GP Presentation Study data |

Health services are defined as follows: In-person GP consultation – a face-to-face consultation with a GP that takes place at the clinic; Telephone GP consultation – a consultation with a GP that takes place over the telephone; Out-of-hours consultation – a consultation with a dedicated service that provides health services outside GP operating hours (evenings and weekends); Accident & Emergency visit – a consultation at a hospital Accident & Emergency department; Telephone information and advice line – a telephone call to a dedicated service that provides syndrome-based medical triage and advice (at the time of this study, these services were NHS Direct in England and Wales, and NHS24 in Scotland); Hospital admission – an overnight stay in a hospital as a result of illness.

## Table D: Unit costs of care by health service

| Health service costs | Unit cost | Source | Comments |
| --- | --- | --- | --- |
| Telephone information and advice line | £20.53 | Munro 2001 [5] | Updated to 2008-9 prices using HCHS Pay & Prices Index |
| Out-of-hours consultation | £77.92 | Santos 2009 [6] | Updated to 2008-9 prices using HCHS Pay & Prices Index |
| Phone call to GP | £18.00 | Curtis 2009 [7] |  |
| In-person GP consultation | £31.00 | Curtis 2009 [7] |  |
| Accident & Emergency | £93.00 | Curtis 2009 [7] |  |
| Hospitalisation | £467.09 | NHS reference costs 2008-9 [8] |  |

Health services are defined as follows: In-person GP consultation – a face-to-face consultation with a GP that takes place at the clinic; Telephone GP consultation – a consultation with a GP that takes place over the telephone; Out-of-hours consultation – a consultation with a dedicated service that provides health services outside GP operating hours (evenings and weekends); Accident & Emergency visit – a consultation at a hospital Accident & Emergency department; Telephone information and advice line – a telephone call to a dedicated service that provides syndrome-based medical triage and advice (at the time of this study, these services were NHS Direct in England and Wales, and NHS24 in Scotland); Hospital admission – an overnight stay in a hospital as a result of illness.

## Table E: Cost to patients by pathogen (IID1 Study) [9]

|  | Cost per case | | |
| --- | --- | --- | --- |
| Cases not seeking medical care | *Campylobacter* | Norovirus | Rotavirus |
| Direct costs to patients^1^ | £4.20 | £2.18 | £0.00 |
| Loss of employment in cases^2^ | £23.97 | £0.00 | £0.00 |
| Loss of employment for carers^2^ | £0.00 | £12.20 | £0.00 |
| Total (1995 prices) | £28.17 | £14.38 | £0.00 |
| **Total adjusted to 2008-9 prices** | **£30.99** | **£15.82** | **£0.00** |
|  |  |  |  |
| Cases seeking medical care | *Campylobacter* | Norovirus | Rotavirus |
| Direct costs to patients^1^ | £17.80 | £12.10 | £17.07 |
| Loss of employment in cases^2^ | £214.32 | £78.94 | £23.58 |
| Loss of employment for carers^2^ | £32.46 | £48.13 | £76.63 |
| Total (1995 prices) | £264.58 | £139.17 | £117.28 |
| **Total adjusted to 2008-9 prices** | **£379.61** | **£199.68** | **£168.27** |

^1^ Includes cost of medications, telephone calls to health services, purchase of goods as a result of illness (foods, entertainment, clothing, bedding, cleaning products), and costs incurred as a result of cancelled activities or childcare. ^2^ Costs associated with time taken off work by patients and their carers are based on reported occupations, number of days off work, and valued according to the New Earnings Survey (1996). Estimation methods are described in [2,9].

## Figure A: Fitted Beta distributions for *Campylobacter*-related health service usage


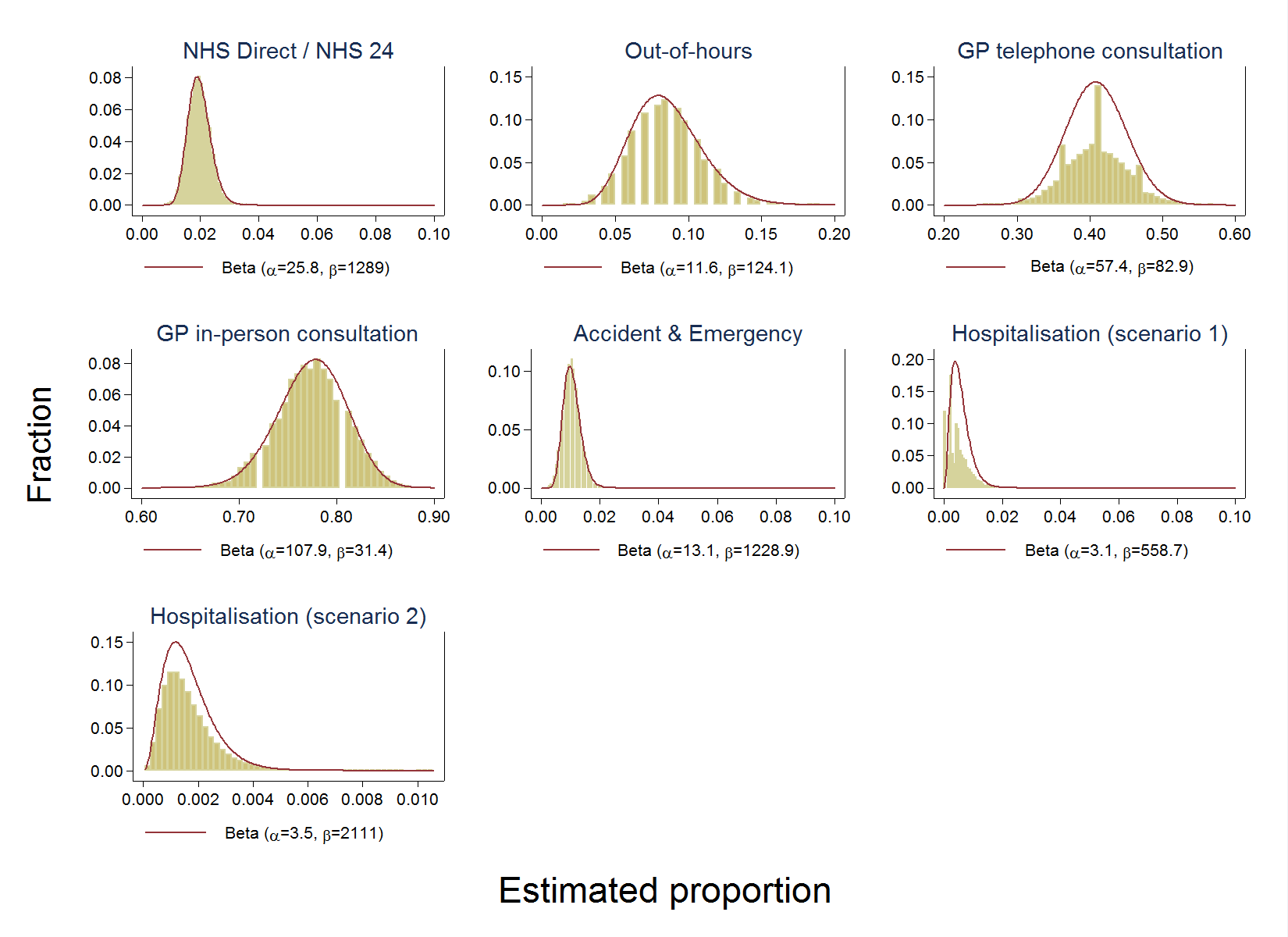


## Figure B: Fitted Beta distributions for norovirus-related health service usage


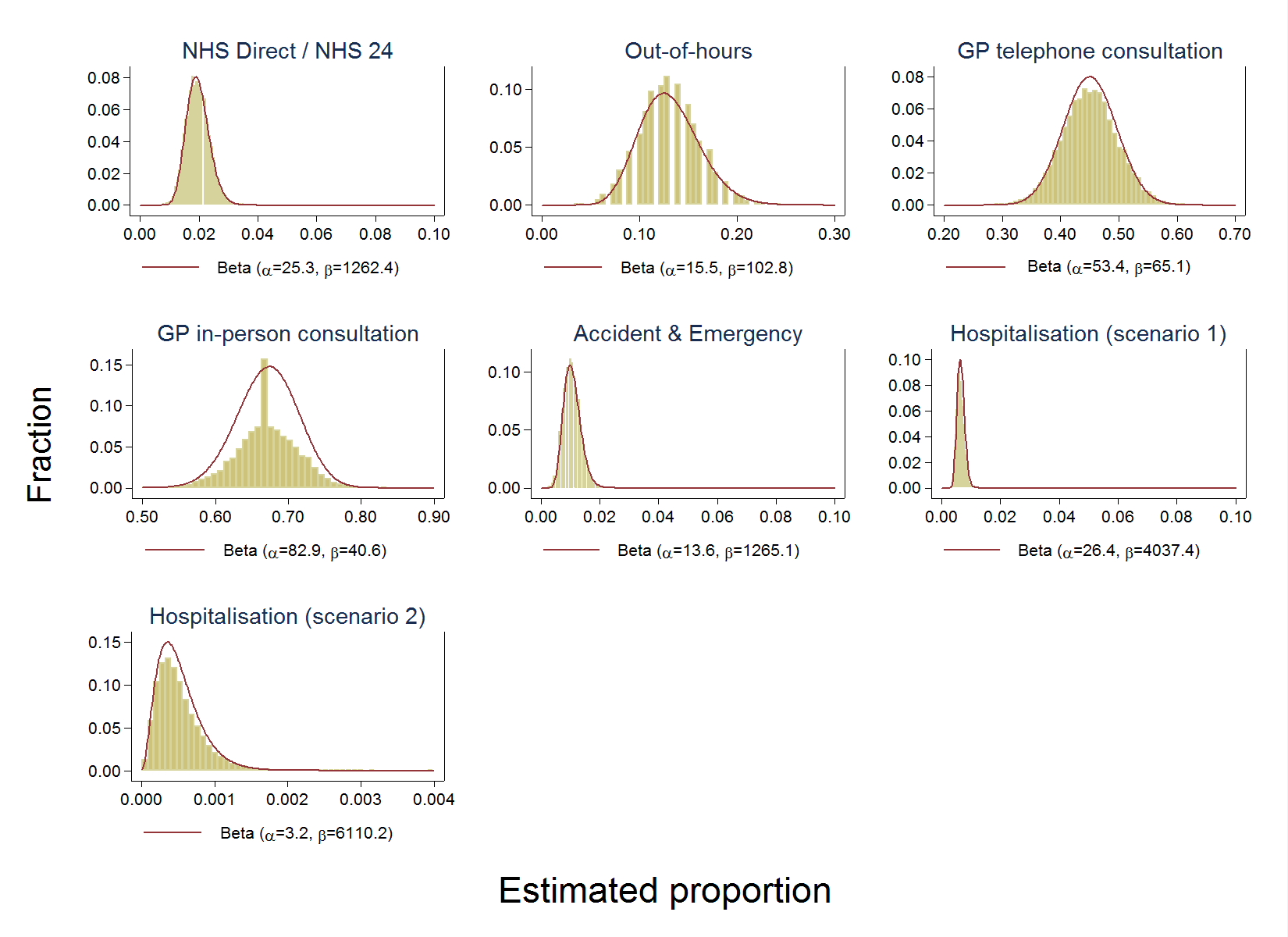


## Figure C: Fitted Beta distributions for rotavirus-related health service usage


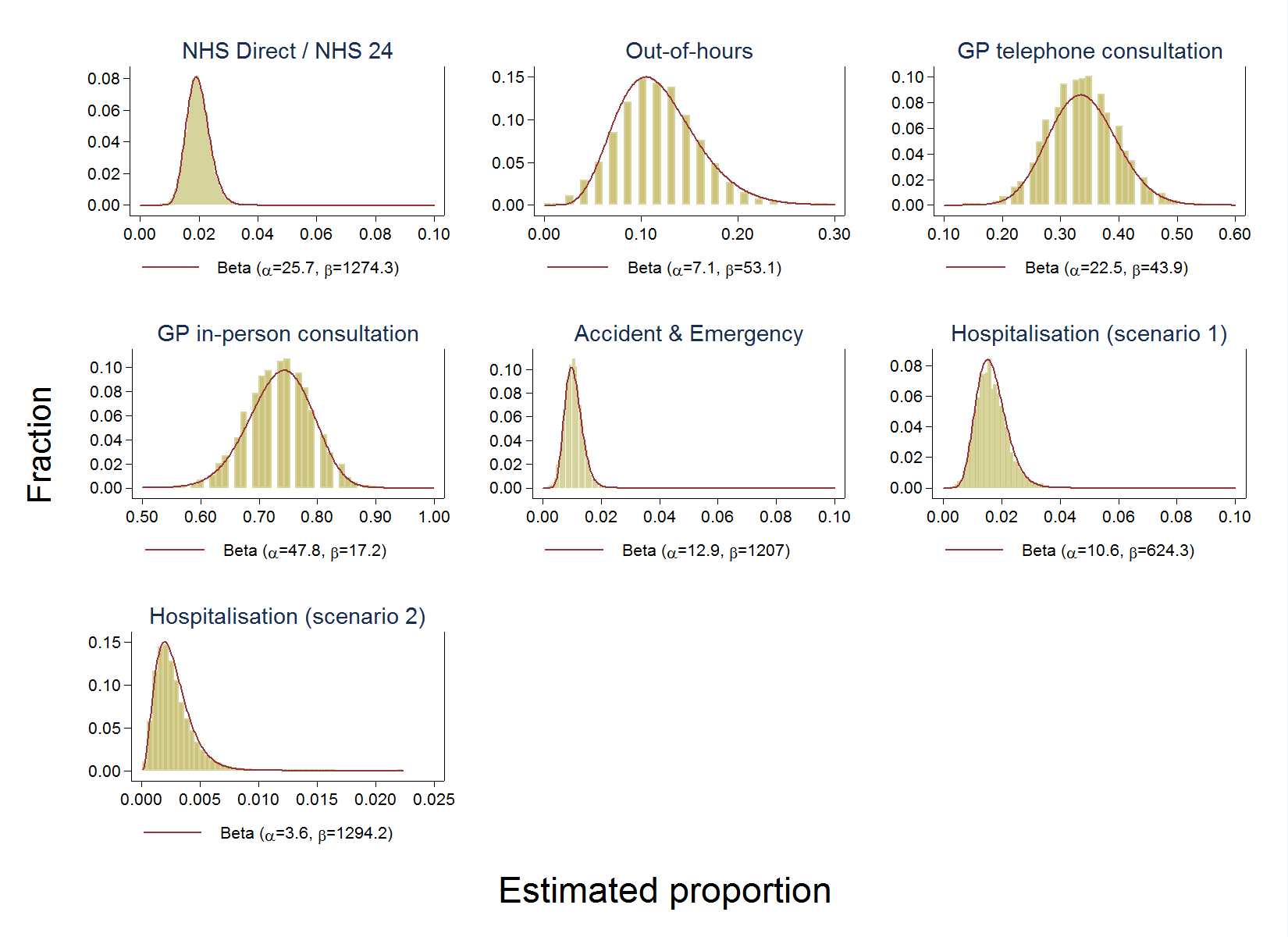


## References:

1. Tam CC, Rodrigues LC, Viviani L, Dodds JP, Evans MR, Hunter PR, et al. Longitudinal study of infectious intestinal disease in the UK (IID2 study): incidence in the community and presenting to general practice. Gut. BMJ Group; 2012;61: 69–77.

2. Roberts JA, Cumberland P, Sockett PN, Wheeler J, Rodrigues LC, Sethi D, et al. The study of infectious intestinal disease in England: socio-economic impact. Epidemiol Infect. 2003;130: 1–11. Available: http://www.pubmedcentral.nih.gov/articlerender.fcgi?artid=2869933&tool=pmcentrez&rendertype=abstract

3. Wall PG, de Louvois J, Gilbert RJ, Rowe B. Food poisoning: notifications, laboratory reports, and outbreaks--where do the statistics come from and what do they mean? Commun Dis Rep CDR Rev. 1996;6: R93–100. Available: http://www.ncbi.nlm.nih.gov/pubmed/8680502

4. Tam CC, Rodrigues LC, Petersen I, Islam A, Hayward A, O’Brien SJ, et al. Incidence of Guillain-Barre syndrome among patients with Campylobacter infection: a general practice research database study. J Infect Dis. Oxford University Press; 2006;194: 95–97.

5. Munro J, Nicholl J, Cathain AO, Knowles E, Morgan A. Evaluation of NHS Direct first wave sites : Final report of the phase 1 research [Internet]. 2001. Available: https://www.shef.ac.uk/polopoly_fs/1.43645!/file/nhsd3.pdf

6. Santos AC, Roberts JA, Cook AJC, Simons R, Sheehan R, Lane C, et al. Salmonella Typhimurium and Salmonella Enteritidis in England: costs to patients, their families, and primary and community health services of the NHS. Epidemiol Infect. 2011;139: 742–53. doi:10.1017/S0950268810001615

7. Curtis L. Unit Costs of Health and Social Care 2009 rev. 1 [Internet]. 2009. Available: http://www.pssru.ac.uk/project-pages/unit-costs/2009/

8. Department of Health. NHS Reference Costs 2008-9 [Internet]. 2009. Available: http://data.gov.uk/dataset/nhs-reference-costs2008-09

9. Food Standards Agency. A Report of the Study of Infectious Intestinal Disease in England [Internet]. London; 2000. Available: http://www.esds.ac.uk/doc/4092%5Cmrdoc%5Cpdf%5C4092userguide6.pdf
