# Supplementary material for: Economic Cost of Campylobacter, Norovirus and Rotavirus Disease in the United Kingdom
Source: PLoS One. 2016 Feb 1;11(2):e0138526. doi: 10.1371/journal.pone.0138526 (PMC4735491; doi:10.1371/journal.pone.0138526)
Supplement: S2 File — (DOC) [file pone.0138526.s002.doc]

Additional file 1

#### EVEREST Statement: Checklist for health economics paper

|  | Study section | Additional remarks |
| --- | --- | --- |
| Study design |  |  |
| (1) The research question is stated | Methods, para 1 |  |
| (2) The economic importance of the research question is stated | Introduction |  |
| (3) The viewpoint(s) of the analysis are clearly stated and justified | Methods, para 2 |  |
| (4) The rationale for choosing the alternative programmes or interventions compared is stated | N/A | This study does not compare alternative interventions |
| (5) The alternatives being compared are clearly described | N/A |  |
| (6) The form of economic evaluation used is stated | Methods |  |
| (7) The choice of form of economic evaluation is justified in relation to the questions addressed | Methods |  |
|  |  |  |
| Data collection |  |  |
| (8) The source(s) of effectiveness estimates used are stated | N/A | This study does not compare alternative interventions |
| (9) Details of the design and results of effectiveness study are given (if based on single study) | N/A | This study does not compare alternative interventions |
| (10) Details of the method of synthesis or meta-analysis of estimates are given (if based on an overview of a number of effectiveness studies) | N/A | This study does not compare alternative interventions |
| (11) The primary outcome measure(s) for the economic evaluation are clearly stated | Methods, para 1, Cost model |  |
| (12) Methods to value health states and other benefits are stated | N/A |  |
| (13) Details of the subjects from whom valuations were obtained are given | N/A |  |
| (14) Productivity changes (if included) are reported separately | N/A |  |
| (15) The relevance of productivity changes to the study question is discussed | N/A |  |
| (16) Quantities of resources are reported separately from their unit costs | Methods, Results, Table 1 |  |
| (17) Methods for the estimation of quantities and unit costs are described | Methods, Cost model, Online Appendix |  |
| (18) Currency and price data are recorded | Methods, Cost data, Table 1 |  |
| (19) Details of currency of price adjustments for inflation or currency conversion are given | Methods, Online Appendix |  |
| (20) Details of any model used are given | Methods, Online Appendix |  |
| (21) The choice of model used and the key parameters on which it is based are justified | Methods, Online Appendix |  |
|  |  |  |
| Analysis and interpretation of results |  |  |
| (22) Time horizon of costs and benefits is stated | Introduction, Methods, Table 1 |  |
| (23) The discount rate(s) is stated | N/A |  |
| (24) The choice of rate(s) is justified | N/A |  |
| (25) An explanation is given if costs or benefits are not discounted | N/A |  |
| (26) Details of statistical tests and confidence intervals are given for stochastic data | Methods, Cost model, Online Appendix |  |
| (27) The approach to sensitivity analysis is given | Methods, Hospital admissions |  |
| (28) The choice of variables for sensitivity analysis is justified | Methods, hospital admissions |  |
| (29) The ranges over which the variables are varied are stated | Table 1 |  |
| (30) Relevant alternatives are compared | N/A |  |
| (31) Incremental analysis is reported | N/A |  |
| (32) Major outcomes are presented in a disaggregated as well as aggregated form | Table 1, Figures 1 and 2 |  |
| (33) The answer to the study question is given | Results, Discussion |  |
| (34) Conclusions follow from the data reported | Discussion |  |
| (35) Conclusions are accompanied by the appropriate caveats | Discussion |  |
